# The efficacy and safety of liraglutide added to metformin in patients with diabetes: a meta-analysis of randomized controlled trials

**DOI:** 10.1038/srep32714

**Published:** 2016-09-07

**Authors:** Jianqiu Gu, Xin Meng, Yan Guo, Lei Wang, Hongzhi Zheng, Yixuan Liu, Bingshu Wu, Difei Wang

**Affiliations:** 1 Department of Endocrinology, the First Affiliated Hospital of China Medical University, China; 2 Department of Geratology, the First Affiliated Hospital of China Medical University, China

## Abstract

Liraglutide, a glucagon-like peptide (GLP-1) receptor agonist, has showed favorable effects in the glycaemic control and weight reduction in patients with type 2 diabetes mellitus (T2DM). The meta-analysis was to compare the efficacy and safety of liraglutide added to metformin with other treatments in patients with T2DM. A systematic literature search on PubMed, Embase, Web of Science and the Cochrane library databases were performed. Eligible studies were randomized controlled trials (RCTs) of patients with T2DM who received the combination treatment of liraglutide and metformin. Pooled estimates were performed using a fixed-effects model or random-effects model. A total of nine RCTs met the inclusion criteria. Compared with control (placebo, sitagliptin, glimepiride, dulaglutide, insulin glargine, and NPH), liraglutide in combination with metformin resulted in significant reductions in HbA1c, bodyweight, FPG, and PPG, and similar reductions in SBP, and DBP. Moreover, liraglutide combined with metformin did not increase the risk of hypoglycemia, but induced a higher incidence of gastrointestinal disorders. In conclusion, this meta-analysis confirmed the use of liraglutide as add-on to metformin appeared to be effective and safe for patients with T2DM. However, considering the potential limitations in this study, more large-scale, well-conducted RCTs are needed to identify our findings.

Type 2 diabetes mellitus (T2DM) is a complex and progressive multi-system disease characterized by declining beta cell function and insulin resistance, which lead to loss of glycemic control and eventual diabetes complications^1^,^2^. Lifestyle modifications and metformin have been recommended by the American Diabetes Association (ADA) and European Association for the Study of Diabetes (EASD) as the first-line therapy for T2DM^3^. For the currently available therapies, because of the un-addressed issue of declining beta cell function and weight or cardiovascular concerns, the glycaemia are not adequately controlled in the long term. Moreover, such therapies often contain complex treatment and titration regimens, and can result in increased risk of hypoglycaemia and undesirable outcomes such as oedema and weight gain^4^.

Glucagon-like peptide-1 (GLP-1) is a naturally occurring incretin hormone released from neuroendocrine intestinal L-cells^5^. It could reduce the glucose levels by increasing the secretion of insulin and lowering glucagon, delaying gastric emptying, and decreasing food intake^5^,^6^. In animal models, native GLP-1 showed beneficial effects in the stimulation of beta cell proliferation and suppression of apoptosis *in vitro*^7^. Moreover,GLP-1 also reduces the risk of hypoglycaemia since its activities in the glucose-lowering are glucose dependent^8^.However, GLP-1 is rapidly metabolized and inactivated by the protease dipetidyl peptidase-4 (DPP-4)^9^, which leads to a very short half-life and limits its therapeutic potential in the clinical practice^10^. The short half life of GLP-1 has prompted efforts to develop novel agents for the therapeutic applications.

Liraglutide is a novel, long-acting GLP-1receptor agonist that is administered once-daily for the treatment of T2DM^11^. It has been approved by the European Union regulatory agency and United States Food and Drug Administration (FDA). Liraglutide has a half-life of 13 h, which allows for once-daily dosing subcutaneous administration^12^,^13^,^14^. The efficacy and safety of liraglutide have been extensively evaluated in several large-scale clinical trials, and their results suggest its positive effects in the reduction of blood glucose, body weight and systolic blood pressure (SBP)^15^. Liraglutide also was generally tolerated and was associated with most commonly adverse events, including transient nausea, vomiting, and diarrhea^16^,^17^,^18^,^19^,^20^.

In this study we conducted a meta-analysis to compare the efficacy and safety of liraglutide plus metformin with other drugs in patients with T2DM.

## Methods and Materials

### Search strategy

We conducted this meta-analysis of the current literature according to the Preferred Reporting Items for Systematic Reviews and Meta-Analyses (PRISMA) guidelines^21^. A comprehensive systematic search of several major electronic databases (PubMed, Embase, Web of Science and the Cochrane library) was conducted before February 3, 2016. The following search terms used were: (“diabetes mellitus, type 2”[MeSH Terms] OR “type 2 diabetes mellitus”[All Fields] OR “type 2 diabetes”[All Fields]) AND (“metformin”[MeSH Terms] OR “metformin”[All Fields]) AND (“liraglutide”[MeSH Terms] OR “liraglutide”[All Fields]). Additional relevant articles were obtained by searching the reference lists of the articles included in this study. No language restriction and publication status were imposed.

### Inclusion criteria and study selection

All clinical trials assessing the efficacy and safety of liraglutide plus metformin in the treatment of T2DM were considered eligible for analysis. The predetermined study inclusion criteria were: (1) randomized controlled trials (RCTs); (2) adult patients had T2DM [HbA1c between either 6.5 or 7.0 and either 10.0 or 11.0%, depending on previous treatment]; (3) compared liraglutide in addition to metformin with another antidiabetic therapy or placebo; (4) reported the data on changes from baseline in HbA1c, bodyweight, fasting plasma glucose (FPG), postprandial plasma glucose(PPG), systolic blood pressure (SBP), and diastolic blood pressure (DBP); (5) treat patients more than 12 weeks after randomization.

### Data extraction and quality assessment

Two independent investigators used a standardized tool to extract the following data from each study: first author’s name, year of publication, country, number of study patients, baseline patient characteristics (age, sex, race, diabetes duration), mean changes from baseline in HbA1c, bodyweight, FPG, PPG, SBP, and DBP, and incidence of treatment-emergent adverse events (nausea, diarrhea, vomiting, dyspepsia, constipation, and nasopharyngitis).

We used the Jadad scale^22^ to assess the methodological quality of the included studies. The Jadad scale consists of three items describing randomization (0–2 points), blinding (0–2 points), and dropouts and withdraws (0–1 point) to report the quality of a RCT^22^. A score of 1 point is given for each of the points described. A further point is obtained when the randomization and/or blinding is described and appropriate. The quality scale ranges from 0 to 5 points, and higher scale suggests better reporting. Any study with a score ≥3 is considered to be of high quality^22^,^23^.

### Statistical analysis

Data were analyzed using Stata version 12.0 (Stata Corporation, College Station, TX, USA). Before the data were synthesized, we first test the heterogeneity between the studies using Q chi-square test^24^, in which a P value <0.10 was considered as significant heterogeneity. ***I***^2^ statistic was used to describe the percentage of the variability that attributed to heterogeneity across the studies rather than the chance. Studies with an ***I***^2^ statistic of <25%, ~50%, ~75%, ~100% are considered to have no, low, moderate, and high degree of heterogeneity, respectively^25^. Pooled estimates were calculated using a fixed-effects model (Mantel–Haenszel method)^26^; otherwise, a random-effects model (DerSimonian–Laird method)^27^ was applied when significant heterogeneity among the included studies was found. If the heterogeneity was tested, subgroup analysis or sensitivity analysis was performed to explore the potential sources of heterogeneity.

Continuous variables, including mean changes from baseline in HbA1c, bodyweight, FPG, PPG, SBP, and DBP, were expressed as weight mean difference (WMD) with 95% confidence intervals (95%CIs); dichotomous variables, including the incidence of treatment-emerge adverse events, were expressed as relative risk (RR) with 95%CIs. The assessment of publication bias was evaluated by using Egger^28^ and Begger^29^ test. A *P* value less than 0.05 was judged as statistically significant, except where otherwise specified.

## Results

### Identification of eligible studies

**Figure 1:**
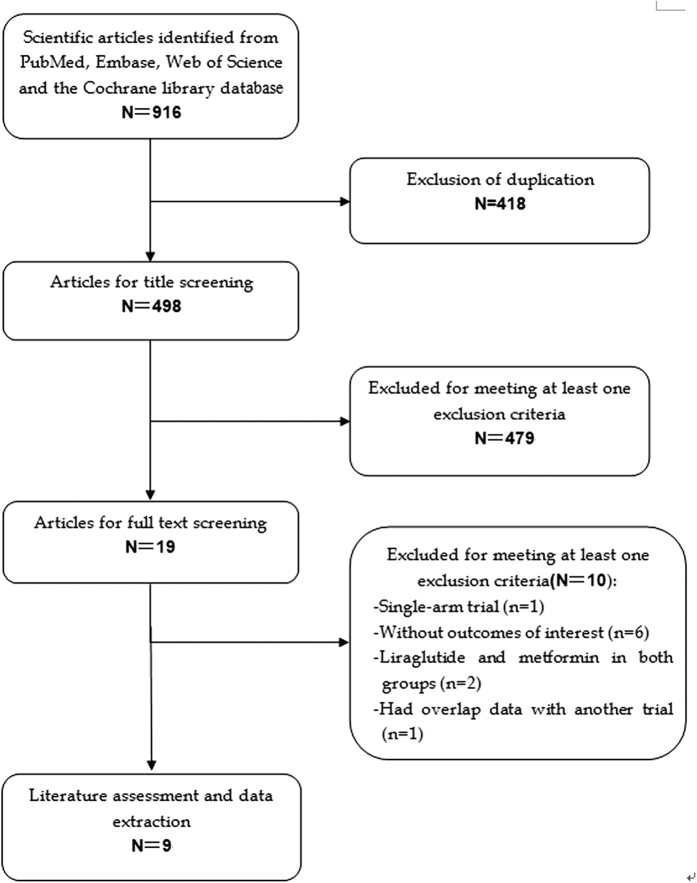
Eligibility of studies for inclusion in meta-analysis.

The initial search yielded 916 relevant publications from PubMed, Web of Science, Embase, and the Cochrane library. Of these, 418 were excluded because of duplicate records, and 479 and 9 studies were removed after a review if title/abstract and full-text information, respectively (Fig. 1). Thus, 19 potential studies were identified for the final analysis. However, ten of them were excluded for the following reasons: six studies did not provide outcome of interest or available data^30^,^31^,^32^,^33^,^34^,^35^, two studies had liraglutide and metformin in both groups^36^,^37^, one study was a sing-arm design^38^, and one study^39^ had overlap data with another trial^40^. Finally, nine RCTs^19^,^20^,^40^,^41^,^42^,^43^,^44^,^45^,^46^ met the inclusion criteria, and were included in this meta-analysis.

### Characteristics of eligible studies and quality assessment

The main characteristics of the nine included RCTs are presented in Table 1. These studies were published between 2009 and 2015. The total number of included patients was 4,657, ranging from 63 to 1,091 patients per study. The clinical characteristics were well matched for age, sex distribution, duration of diabetes, BMI, waist circumference, HbA1c, FPG, SBP, and DBP in both groups at the beginning of each study. The study durations ranged from 12 to 52 weeks. All these studies provided information regarding the efficacy and safety of combination treatment of liraglutide and metformin in patients with T2DM.

The dosages of liraglutide were fixed in eight trials^19^,^20^,^40^,^41^,^42^,^43^,^44^,^45^ with a dose of 0.6, 1.2 or 1.8 mg/day, whereas the other study^46^ started at a dose of 0.6 mg/day, up-titrated to 1.2 mg/day after 1 week. Placebo was used as control group in two studies^19^,^20^, four used 100 mg/day sitagliptin^40^,^43^,^46^, two used 4 mg/day glimepiride^19^,^45^,one used 1.5 mg/week dulaglutide^41^, one used 24 IU/day insulin glargine^20^, one used NPH^42^. The median Jadad score of these included studies was 4 (ranged from 3 to 4).

### Change in HbA1c

**Figure 2:**
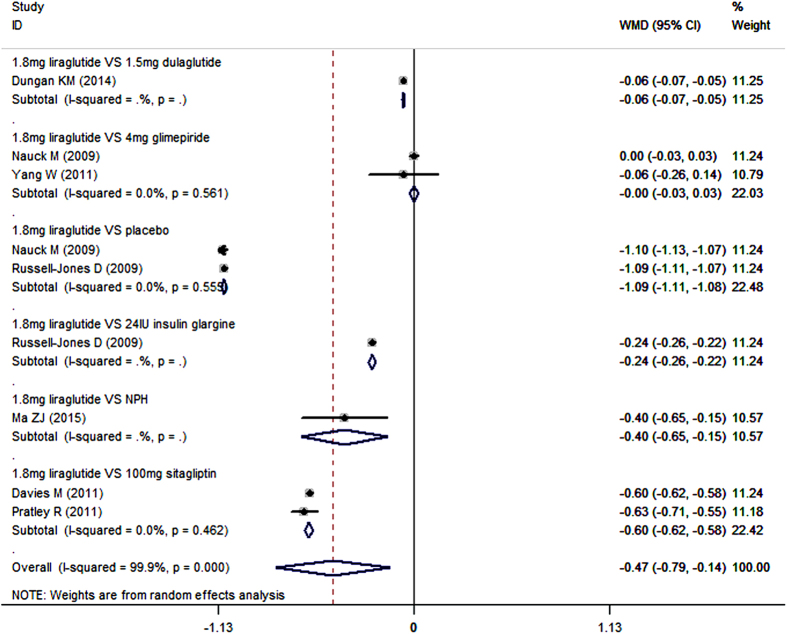
HbA1c: 1.8 mg liraglutide add-on to metformin VS. control.

**Figure 3:**
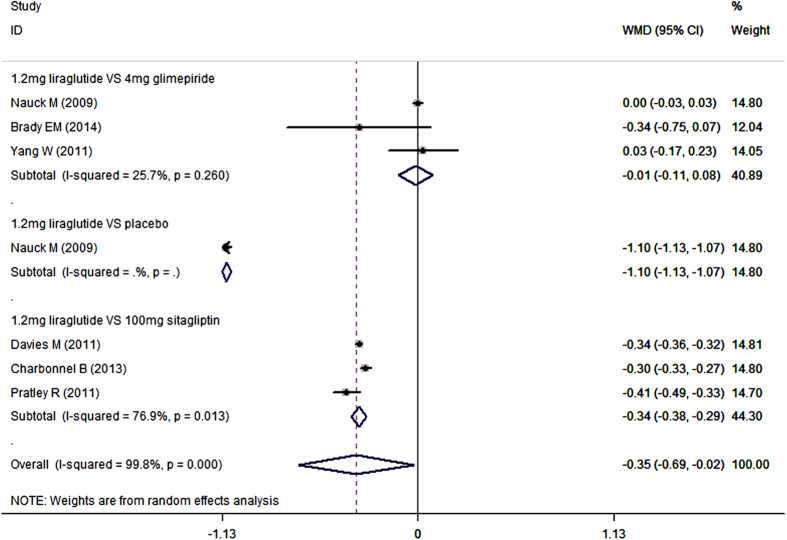
HbA1c: 1.2 mg liraglutide add-on to metformin VS. control.

All the studies included reported the data of mean change from baseline in HbA1c^19^,^20^,^40^,^41^,^42^,^43^,^44^,^45^,^46^. When used as add-on to metformin, liraglutide significantly decreased HbA1c compared with control (placebo, sitagliptin, glimepiride, dulaglutide, insulin glargine, and NPH) (WMD = −0.36%, 95%CI: −0.57%, −0.14%; P = 0.001). Subgroup analysis based on the dosage of liraglutide showed that, 1.8 mg/day and 1.2 mg/day liraglutide in combination with metformin notably lowered HbA1c compared with control (for 1.8 mg/day liraglutide: WMD = −0.47%, 95%CI: −0.79%, −0.14%; P = 0.005; for 1.2 mg/day liraglutide: WMD = −0.35%, 95%CI: −0.69%, −0.02%; P = 0.035)(Figs 2 and 3), whereas 0.6 mg/day liraglutide did not (WMD = −0.09%, 95%CI: −0.96%, 0.79%; P = 0.848).

Furthermore, we also conducted subgroup-analysis based on the comparators. As an add-on to metformin, 1.8 mg/day liraglutide significantly lowered HbA1c compared with placebo (WMD = −1.09%, 95%CI: −1.11%, −1.08%; P < 0.001), sitagliptin (WMD = −0.60%, 95%CI: −0.62%, −0.58%; P < 0.001), dulaglutide (WMD = −0.06%, 95%CI: −0.07%, −0.05%; P < 0.001), and insulin glargine (WMD = −0.24%, 95%CI: −0.26%, −0.22%; P < 0.001), but not glimepiride (WMD = 0.00%, 95%CI: −0.03%, 0.03%; P = 0.936) (Fig. 2) (Table 2).

When used combination with metformin, 1.2 mg/day liraglutide notably decreased HbA1c compared with placebo (WMD = −1.10%, 95%CI: −1.13%, −1.07%; P < 0.001), and sitagliptin (WMD = −0.28%, 95%CI: −0.47%, −0.10%; P < 0.001), but not glimepiride (WMD = −0.01%, 95%CI: −0.11%, 0.08%; P = 0.807) (Fig. 3) (Table 2).

When used as an add-on therapy to metformin, 0.6 mg/day liraglutide notably decreased HbA1c compared with placebo (WMD = −0.80%, 95%CI: −0.83%, −0.77%; P < 0.001), but increased HbA1c compared with glimepiride (WMD = 0.30%, 95%CI: 0.27%, 0.33%; P < 0.001) (Table 2).

### Bodyweight

**Figure 4:**
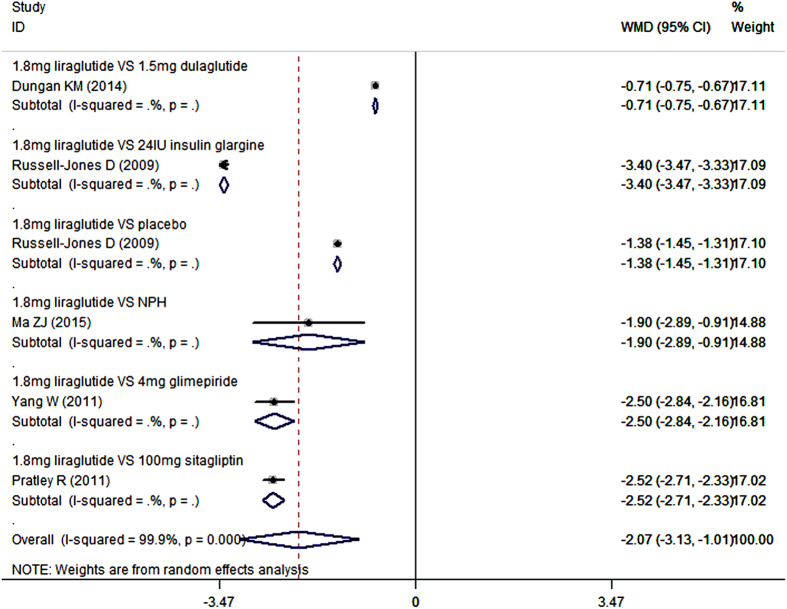
Bodyweight: 1.8 mg liraglutide add-on to metformin VS. control.

**Figure 5:**
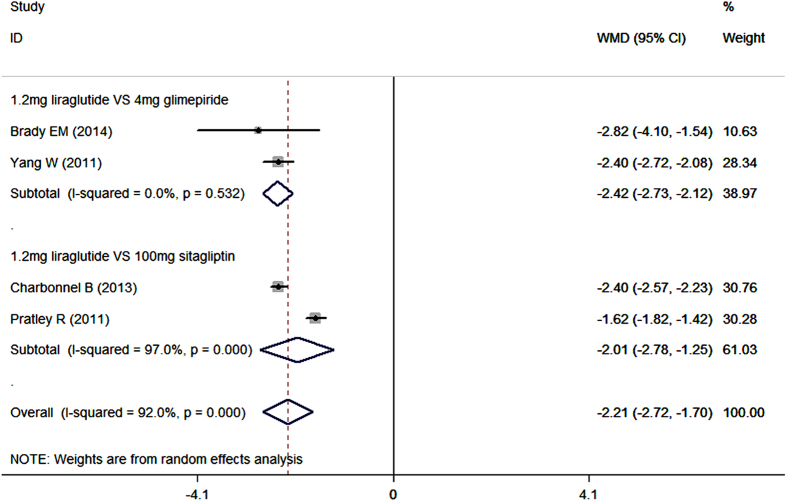
Bodyweight: 1.2mg liraglutide add-on to metformin VS. control.

Seven studies reported the data in bodyweight^20^,^40^,^41^,^42^,^44^,^45^,^46^. As an add-on to metformin, liraglutide lowered bodyweight more than control (placebo, sitagliptin, glimepiride, dulaglutide, insulin glargine, and NPH) (WMD = −2.13 kg, 95%CI: −2.87, −1.38; P < 0.001). Subgroup analysis based on the dosage of liraglutide showed that, liraglutide in all of the three dosages significantly reduced bodyweight more than control when combined with metformin (for 1.8 mg/day: WMD = −2.07 kg, 95%CI: −3.13, −1.01; P < 0.001; for 1.2 mg/day: WMD = −2.21 kg, 95%CI: −2.72, −1.70; P < 0.001; for 0.6 mg/day: WMD = −1.90 kg, 95%CI: −2.19, −1.61; P < 0.001) (Figs 4 and 5).

Furthermore, we also conducted subgroup-analysis based on the comparators. As an add-on to metformin, 1.8 mg/day liraglutide significantly lowered bodyweight compared with placebo (WMD = −1.38 kg, 95%CI: −1.45, −1.31; P < 0.001), sitagliptin (WMD = −2.52 kg, 95%CI: −2.71, −2.33; P < 0.001), glimepiride (WMD = −2.50 kg, 95%CI: −2.84, −2.16; P < 0.001), dulaglutide (WMD = −0.71 kg, 95%CI: −0.75, −0.67; P < 0.001), insulin glargine (WMD = −3.40 kg, 95%CI: −3.47, −3.33; P < 0.001), and NPH (WMD = −1.90 kg, 95%CI: −2.89, −0.91; P < 0.001) (Fig. 4) (Table 2).

When used combination with metformin, 1.2 mg/day liraglutide notably decreased bodyweight compared with sitagliptin (WMD = −2.01 kg, 95%CI: −2.78, −1.25; P < 0.001), and glimepiride (WMD = −2.42 kg, 95%CI: −2.73, −2.12; P < 0.001) (Fig. 5) (Table 2).

### Fasting plasma glucose

**Figure 6:**
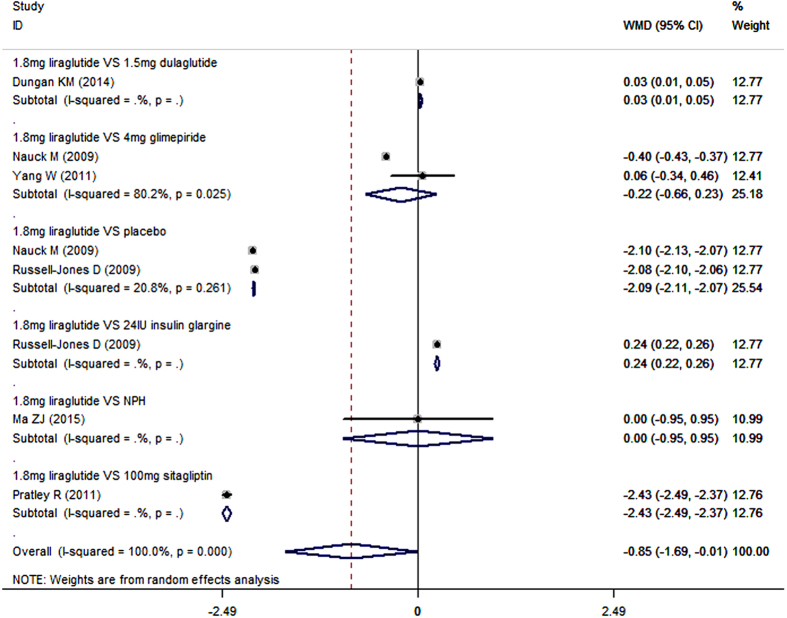
Fasting plasma glucose: 1.8 mg liraglutide add-on to metformin VS. control.

**Figure 7:**
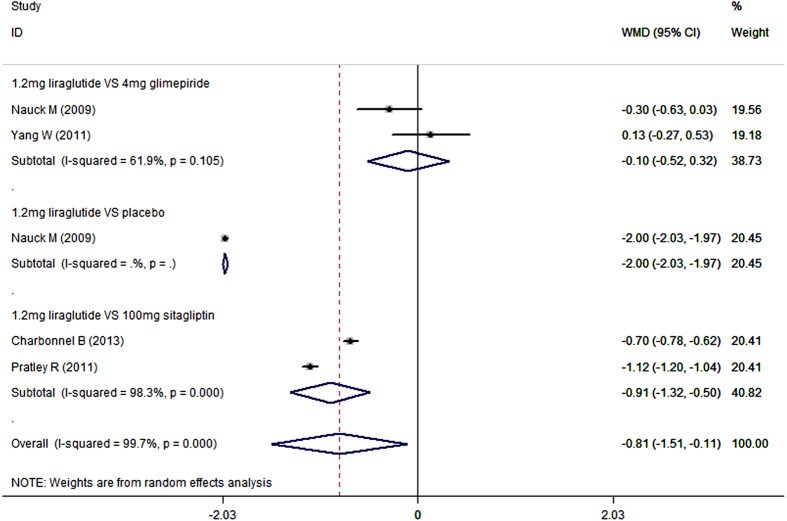
Fasting plasma glucose: 1.2 mg liraglutide add-on to metformin VS. control.

Seven studies reported the data in FPG^19^,^20^,^40^,^41^,^42^,^45^,^46^. When administered with metformin, liraglutide significantly lowered the FPG concentration compared with control (WMD = −0.72 mmol/L, 95%CI: −1.27, −0.17; P = 0.010). Subgroup analysis based on the dosage of liraglutide showed that, 1.8 mg/day and 1.2 mg/day liraglutide in combination with metformin notably reduced the FPG concentration compared with control (for 1.8 mg/day: WMD = −0.85 mmol/L, 95%CI: −1.69, −0.10; P = 0.046; for 1.2 mg/day: WMD = −0.81 mmol/L, 95%CI: −1.51, −0.11; P = 0.023) (Figs 6 and 7), whereas 0.6 mg/day liraglutide did not (WMD = −0.65 mmol/L, 95%CI: −2.32, 1.01; P = 0.441).

Furthermore, we also conducted subgroup-analysis based on the comparators. As an add-on to metformin, 1.8 mg/day liraglutide decreased FPG concentration more than placebo (WMD = −2.09 mmol/L, 95%CI: −2.11, −2.07; P < 0.001) and sitagliptin (WMD = −2.43 mmol/L, 95%CI: −2.49, −2.37; P < 0.001), and reduced FPG concentration less than dulaglutide (WMD = 0.03 mmol/L, 95%CI: 0.01, 0.05; P = 0.002) and insulin glargine (WMD = 0.24 mmol/L, 95%CI: 0.22, 0.26; P < 0.001), and exhibited a similar reduction in FPG concentration as did glimepiride (WMD = −0.22 mmol/L, 95%CI: −0.66, 0.23; P = 0.340) (Fig. 6) (Table 2).

When used combination with metformin, 1.2 mg/day liraglutide notably decreased FPG concentration more than placebo (WMD = −2.00 mmol/L, 95%CI: −2.03, −1.97; P < 0.001) and sitagliptin (WMD = −0.91 mmol/L, 95%CI: −1.32, −0.05; P < 0.001), and exhibited a similar reduction in FPG concentration as did glimepiride (WMD = −0.10 mmol/L, 95%CI: −0.52, 0.32; P = 0.641) (Fig. 7) (Table 2).

When used as an add-on therapy to metformin, 0.6 mg/day liraglutide notably reduced FPG level compared with placebo (WMD = −1.50 mmol/L, 95%CI: −1.53, −1.47; P < 0.001), and exhibited a similar reduction in FPG concentration as did glimepiride (WMD = 0.20 mmol/L, 95%CI: −0.05, 0.45; P = 0.116) (Table 2).

### Postprandial plasma glucose

**Figure 8:**
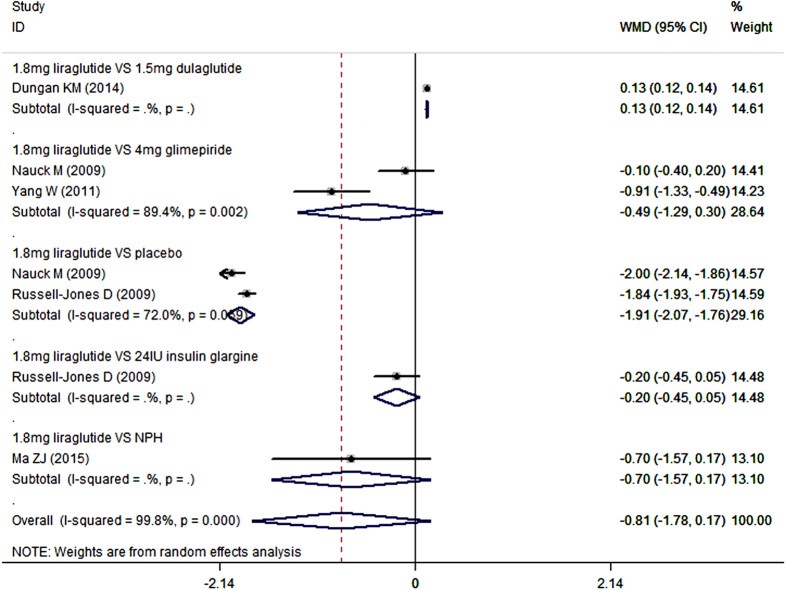
Postprandial plasma glucose: 1.8 mg liraglutide add-on to metformin VS. control.

**Figure 9:**
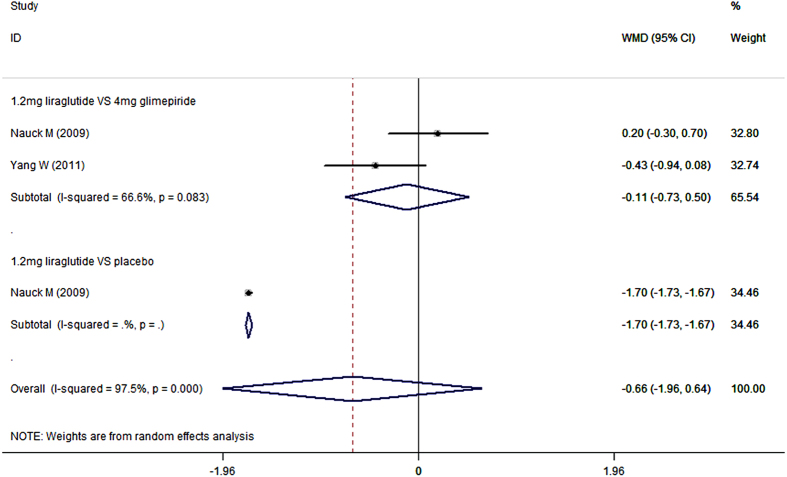
Postprandial plasma glucose: 1.2 mg liraglutide add-on to metformin VS. control.

Five studies reported the data in PPG concentration^19^,^20^,^41^,^42^,^45^. When administered with metformin, liraglutide showed more reduction in PPG than control (placebo, glimepiride, insulin glarigin, dulaglutide, and NPH) (WMD = −0.60 mmol/L, 95%CI: −1.17, −0.03; P < 0.001). Subgroup analysis based on the dosage of liraglutide showed that, liraglutide in all of the three dosage resulted in similar change in PPG as did control (for 1.8 mg/day: WMD = −0.81 mmol/L, 95%CI: −1.78, 0.17; P = 0.107; for 1.2 mg/day: WMD = −0.66 mmol/L, 95%CI: −1.96, 0.64; P = 0.320; for 0.6 mg/day: WMD = −0.08 mmol/L, 95%CI: −1.60, 1.44; P = 0. 920) (Figs 8 and 9).

Furthermore, we also conducted subgroup-analysis based on the comparators. As an add-on to metformin, 1.8 mg/day liraglutide decreased PPG concentration more than placebo (WMD = −1.91 mmol/L, 95%CI: −2.07, −1.76; P < 0.001), less than dulaglutide (WMD = 0.13 mmol/L, 95%CI: 0.12, 0.14; P < 0.001), and similar with glimepiride (WMD = −0.49 mmol/L, 95%CI: −1.29, 0.30; P = 0.224), insulin glarigin (WMD = −0.20 mmol/L, 95%CI: −0.45, 0.05; P = 0.112), and NPH (WMD = −0.70 mmol/L, 95%CI: −1.57, 0.17; P = 0.114) (Fig. 8) (Table 2).

When used combination with metformin, 1.2 mg/day liraglutide notably decreased PPG concentration compared with placebo (WMD = −1.70 mmol/L, 95%CI: −1.73, −1.67; P < 0.001), and resulted in similar change in PPG concentration as did glimepiride (WMD = −0.11 mmol/L, 95%CI: −0.73, 0.50; P = 0.720) (Fig. 9) (Table 2).

When used as an add-on therapy to metformin, 0.6 mg/day liraglutide notably reduced PPG concentration compared with placebo (WMD = −1.10 mmol/L, 95%CI: −1.13, −1.07; P < 0.001), and exhibited a similar reduction in PPG concentration as did glimepiride (WMD = 0.47 mmol/L, 95%CI: −0.25, 1.18; P = 0.200) (Table 2).

### Systolic blood pressure

Five studies reported the data in SBP^20^,^40^,^41^,^44^,^46^. As an add-on to metformin, liraglutide resulted in similar reduction in SBP as did control (placebo, sitagliptin, dulaglutide, insulin glargine, and glimepiride) (WMD = −1.67 mm Hg, 95%CI: −3.67, 0.33; P = 0.102). Subgroup analysis based on the dosage of liraglutide showed that, liraglutide with a dosage of 1.8 mg and 1.2 mg led to similar reduction of SBP compared with control (for 1.8 mg/day: WMD = −1.76 mm Hg, 95%CI: −4.79, 1.28; P = 0.256; for 1.2 mg/day: WMD = −1.49 mm Hg, 95%CI: −4.59, 1.61; P = 0.345).

Furthermore, we also conducted subgroup-analysis based on the comparators. As an add-on to metformin, 1.8 mg/day liraglutide lowered more SBP than placebo (WMD = −2.60 mm Hg, 95%CI: −2.78, −2.42; P < 0.001) and insulin glargine (WMD = −4.54 mm Hg, 95%CI: −4.57, −4.51; P < 0.001), but less SBP than dulaglutide (WMD = 0.54 mm Hg, 95%CI: 0.43, 0.65; P < 0.001), and similar SBP than sitagliptin(WMD = −0.42 mm Hg, 95%CI: −0.88, 0.04; P = 0.074) (Table 2).

When used combination with metformin, 1.2 mg/day liraglutide resulted in similar reduction of SBP compared with glimepiride (WMD = −3.70 mm Hg, 95%CI: −9.81, 2.41; P = 0.235) and sitagliptin (WMD = −1.08 mm Hg, 95%CI: −4.46, 2.31; P = 0.533) (Table 2).

### Diastolic blood pressure

Four studies reported the data in DBP^40^,^41^,^44^,^46^. When used as add-on to metformin, liraglutide exhibited similar DBP change as did control (sitagliptin, glimepiride, and dulaglutide) (WMD = 0.11 mm Hg, 95%CI: −0.53, 0.74; P = 0.744). Subgroup analysis based on the dosage of liraglutide showed that, both dosage of liraglutide resulted in a similar change in DBP as did control (for 1.8 mg/day: WMD = 0.25 mm Hg, 95%CI: −0.42, 0.93; P = 0.460; for 1.2 mg/day: WMD = −0.28 mm Hg, 95%CI: −1.53, 0.97; P = 0.662).

Furthermore, we also conducted subgroup-analysis based on the comparators. As an add-on to metformin, 1.8 mg/day liraglutide decreased more DBP than dulaglutide (WMD = −0.09 mm Hg, 95%CI: −0.15, −0.03; P = 0.006), but less DBP than sitagliptin (WMD = 0.60 mm Hg, 95%CI: 0.52, 0.68; P < 0.001) (Table 2).

When used combination with metformin, 1.2 mg/day liraglutide resulted in greater reduction in DBP than glimepiride (WMD = −5.53 mm Hg, 95%CI: −9.15, −1.91; P = 0.003), and similar reduction than sitagliptin (WMD = 0.27 mm Hg, 95%CI: −1.04, 1.58; P = 0.687) (Table 2).

### Treatment-related adverse events

Five studies reported the data in treatment-related adverse events^19^,^20^,^40^,^41^,^46^. The most common treatment-related adverse events, including gastrointestinal disorders (nausea, diarrhea, vomiting, dyspepsia, decreased appetite, and constipation), nasopharyngitis, headache, and back pain are listed in Table 3. When used as add-on therapy to metformin, liraglutide significantly increased the risk of gastrointestinal disorders (RR = 1.59, 95%CI: 1.15, 2.19; P = 0.005) compared with control (placebo, dulaglutide, sitagliptin, glimepiride, NPH, and insulin glargine). The incidence of diarrhea was higher in patients treated with combination therapy of metformin and liraglutide than in the control group (RR = 1.98, 95%CI: 1.30, 3.00; P = 0.001).

There were 44 out of 1483 (2.97%) patients in metformin plus liraglutide group and 32 out of 397 (8.06%) patients in the control group (placebo, glimepiride, and NPH) that experienced hypoglycemia. Pooled results showed that, compared with control, metformin plus liraglutide did not increase the risk of hypoglycemia (RR = 0.33, 95%CI: 0.08, 1.44; P = 0.140). No cases of pancreatitis were reported in these included studies.

### Publication bias

Assessment of publication bias was conducted by using Egger’s and Begg test, and results showed that no publication bias existed among the included studies (Egger’s test: t = −1.33, P = 0.201; Begg test: Z = 0.14, P = 0.887).

## Discussion

The addition of GLP-1 receptor agonist is recommended by the ADA and the EASD as a therapeutic option for patients with T2DM whose HbA1c targets are not met or maintained with lifestyle modifications, with consideration of individual patient-related factors^47^. This meta-analysis investigated the efficacy and safety of liraglutide in combination with metformin, compared to other therapies for patients with T2DM. Overall, the results of our study suggest that compared with other therapies, liraglutide in combination with metformin showed greater reduction in terms of HbA1c levels, body weight, FPG, and PPG, and similar changes in SBP and DBP. In addition, when used as add-on therapy to metformin, liraglutide did not increase the risk of hypoglycemia, but induced a higher incidence of gastrointestinal disorders.

To the bests of our knowledge, this is the first comprehensive meta-analysis to compare the efficacy and safety of liraglutide add-on to metformin with other therapies in patients with T2DM. Our results suggest that, compared with control, combined therapy of liraglutide and metformin reduced the HbA1c significantly by −0.36%. This result is consistent across the subgroup analysis based on dosage of liraglutide, in which 1.8 mg and 1.2 mg liraglutide decreased the HbA1c by −0.47%, and −0.35%, respectively. Furthermore, subgroup analysis based on comparators demonstrated that, liraglutide in combination with metformin was associated with a greater reduction of HbA1c than placebo, sitagliptin, insulin glargin, or dulaglutide, and similar change with glimepiride.

The HbA1c reduction from baseline with the combination therapy of liraglutide and metformin in this meta-analysis was in line with a previously published study. In the trial conducted by Pratley RE, *et al*.^39^, 1.2 mg and 1.8 mg liraglutide decreased the HbA1c from baseline by −1.24% and −1.50%, respectively, whereas sitagliptin lowered the HbA1c by −0.90%. The estimated treatment differences (ETD) for 1.2 mg, 1.8 mg liraglutide versus sitagliptin were −0.34% (95%CI: −0.51, −0.61) and −0.60% (95%CI: −0.77, −0.43), respectively^39^, which indicated that combination therapy of liraglutide and metformin resulted a more reduction of HbA1c than sitagliptin. The greater HbA1c reduction of combination therapy versus sitagliptin probably can be explained by the pharmacological concentrations of free liraglutide, whereas physiological concentrations of GLP-1 and glucose dependent insulinotropic polypeptide (GIP) are achieved with sitagliptin. Although the active GLP-1 concentrations are increased by two or three times with dipeptidyl peptidase-4 (DPP-4) inhibitors^48^, the stimulation of GLP-1 receptor activity by liraglutideis estimated to be several times higher than with DPP-4 inhibitors^14^. Moreover, liraglutide has a long half-life (about 13 h), which may be another reason for the increased efficacy^49^. Despite sitagliptin has a similar pharmacokinetic half-life as liraglutide^50^, its effect on the increase of endogenous GLP-1 concentration occurs mainly after meals. Thus, the fasting concentrations of active GLP-1 remain considerably low overnight, so the FPG concentrations with sitagliptin reduced less than liraglutide^50^.

In this meta-analysis, liraglutide used as an add-on to metformin, reduced bodyweight more than other therapies did. This result can be attributed to the increased stimulation of GLP-1 receptor by liraglutide. T2DM increases morbidity and mortality mainly because of the cardiovascular and cerebrovascular disease. Obesity is one of the specific risk factors for the development of diabetes, and cardiovascular disease^51^. Thus, antidiabetic drugs should have favorable effects in the treatment of cardiovascular and cerebrovascular disease, as well as reduce the blood glucose. Among the controls (placebo, dulaglutide, insulin glargine, NPH, glimepiride, and sitagliptin) of the included studies, weight gain was observed in patients treated with insulin glargine (1.6 kg increase), and glimepiride (0.25 kg increase). However, when liraglutide is added to metformin, it significantly reduced the bodyweight (by 2.13 kg) compared with control. This result is in contrast to the data from a phase 3 trail, in which 1.8 mg once-daily liraglutide monotherapy was compared with 10 μg twice-daily exenatide^16^. And liraglutide did not show a greater reduction in bodyweight than exenatide (liraglutide: −3.24 kg VS exenatide: −2.87 kg; ETD, −0.38 kg, 95%: −0.99, 0.23, P = 0.2235)^16^.

With regard to the FPG, our results showed that combined therapy of liraglutide and metformin was associated with a significant decrease on this clinical outcome compared with control (placebo, sitagliptin, glimepiride, dulaglutide, insulin glargine, and NPH). However, this significant FPG reduction was not observed in the subgroup analysis between liraglutide and glimepiride. Compared with glimepiride, 0.6 mg,1.2 mg, and 1.8 mg liraglutide reduced the FPG by 0.20 mmol/L, −0.10 mmol/L, and −0.22 mmol/L, respectively, though the difference was not significant. These results are in accordance with the findings from the LEAD-2 trial^19^. In that trial, liraglutide was found to have similar efficacy in FPG reduction compared to glimepiride. The decrease in FPG from baseline was 1.1 mmol/L, −1.6 mmol/L, and −1.7 mmol/L for the 0.6 mg, 1.2 mg, and 1.8 mgliraglutide, respectively, whereas the corresponding value for glimepiride was −1.3 mmol/L^19^.

For the PPG, we found the pooled results remained confusing. Our finding showed that liraglutide in combination with metformin was associated with a significantly greater reduction in PPG than control (placebo, glimepiride, dulaglutide, insulin glargine, and NPH) (−0.60 mmol/L). However, when it was compared with active comparators, no significant difference was found between them. Compared with glimepiride, 0.6 mg, 1.2 mg, and 1.8 mgliraglutide reduced PPG by 0.47 mmol/L, −0.11 mmol/L, and −0.49 mmol/L, respectively; however, the difference between them was not significant. These findings are in line with what has been observed in the LEAD trial^19^. In the LEAD trial, it has been found that the decreases from baseline in PPG were −1.7 mmol/L, −2.3 mmol/L, and −2.6 mmol/L for the 0.6 mg, 1.2 mg, and 1.8 mg liraglutide respectively, and −2.5 mmol/L for glimepiride. The corresponding ETD for 0.6 mg, 1.2 mg, and 1.8 mg liraglutide versus glimepiride was 0.8 mmol/L, 0.2 mmol/L, and −0.1 mmol/L, respectively. Similar results were also found in the comparison between liraglutide and dulaglutide^41^, insulin glargine^20^, or NPH^42^, which showed a comparable effect in PPG between them. We speculated that due to the strong response in the placebo group, liraglutide showed beneficial effects in PPG as compared with control.

Hypoglycemia is a challenge and obstacle in the treatment of T2DM. In this meta-analysis, combination treatment of liraglutide and metformin did not increase the risk of hypoglycemia (2.97% VS 8.06%) compared with other therapies (placebo, glimepiride, and NPH). Our results were consistent with the findings of a recently published meta-analysis by Zhang L, *et al*.^52^. In that study, another GLP-1 receptor agonist dulaglutide was compared with other antidiabetic drugs for T2DM. Their results indicated that, as a monotherapy, dulaglutide did not increase the risk of hypoglycemia compared with control (placebo, metformin and liraglutide) (7.8% VS 10.6, respectively); as in the combination with OAM and lispro, dulaglutide resulted in a similar incidence of hypoglycemia (24.5% VS 24.5%) compared with control (placebo, sitagliptin, exenatide and liraglutide)^52^.

In this meta-analysis, although the liraglutide in combination with metformin induced a higher incidence of gastrointestinal disorders than control, these adverse events were mainly mild to moderate. The incidence of diarrhea was higher in patients treated with liraglutide than in the control group, which is consistent with results from previous trials^19^,^20^,^39^,^46^. No cases of pancreatitis were reported in these included studies, thus we did not assess the potential association between incretin and pancreatitis. However, the recent epidemiological studies indicated that, incretin-based therapies did not increase the risk of pancreatitis as compared with other diabetes treatments, and general population with T2DM was not a risk factor for the development of pancreatitis^53^.

We admit that there are several potential limitations in this meta-analysis. First, our meta-analysis was conducted based on nine RCTs, and two of them had a relatively small sample size (less than 100). Although all of these included studies were of high-quality (Jadad ≥3), our findings might be overestimated by these small trials since trials with small sample size were more likely to overestimate the treatment effect compared with those larger trials. Second, considerable heterogeneity existed among these included studies. However, it should not be surprising when considering the inclusion criteria for patients, dosage of liraglutide, duration of study, and different comparators. To explore the potential sources of heterogeneity, we conducted subgroup analysis based on the dosage of liraglutide, and comparators. And the issue of heterogeneity was resolved when the data analysis was performed according to the subgroup analysis. Third, our exploration of the effect comparison between liraglutide with active comparators was insufficient because of sparse reporting among the included studies. Therefore, physicians should interpret our findings with caution when applying them into the clinical practice.

In conclusion, our meta-analysis indicated that, liraglutideas added-on to metformin showed greater reduction in HbA1c levels, body weight, FPG, and PPG, and similar change in SBP and DBP compared with other therapies. However, considering the potential limitations in this study, more large-scale, well-conducted RCTs are needed to identify our findings.

## Additional Information

**How to cite this article**: Gu, J. *et al*. The efficacy and safety of liraglutide added to metformin in patients with diabetes: a meta-analysis of randomized controlled trials. *Sci. Rep.* **6**, 32714; doi: 10.1038/srep32714 (2016).

## Figures and Tables

**Table 1:** Baseline characteristics of patients in the trials included in the meta-analysis.

Study	Year	Intervention	No. of patients	Age(mean ± SD, y)	Duration of T2DM (mean ± SD, y)	Hb1Ac (mean ± SD, %)	Weight(mean ± SD, kg)	Jadad score
Nauck M^19^	2009	0.6 mg liraglutide	242	56±11	7±5	8.4±0.9	NR	4
		1.2 mgliraglutide	242	57±9	7±5	8.3±1.0	NR	
		1.8 mg liraglutide	242	57±9	8±5	8.4±1.0	NR	
		4 mg glimepiride	123	57±9	8±5	8.4±1.0	NR	
		Placebo	242	56±9	8±6	8.4±1.1	NR	
Russell-Jones D^20^	2009	1.8 mg liraglutide	232	57.6±9.5	9.2±5.8	8.3±0.9	85.5±19.4	4
		Placebo	115	57.5±9.6	9.4±6.2	8.3±0.9	85.7±16.7	
		24 IU insulin glargine	234	57.5±10.5	9.7±6.4	8.2±0.9	85±17.9	
Dungan KM^41^	2014	1.8 mg liraglutide	300	56.8±9.9	7.3±5.4	8.1±0.8	94.4±19	4
		1.5 mg dulaglutide	299	56.5±9.3	7.1±5.4	8.1±0.8	93.8±18.2	
Ma ZJ^42^	2015	1.8 mg liraglutide	31	51.3±12.5	NR	10.1±1.4	77.3±2.1	3
		NPH	32	52.5±11.7	NR	9.8±1.8	76.7±2.3	
Davies M^43^	2011	1.2 mg liraglutide	164	NR	NR	8.4	93.7	3
		1.8 mg liraglutide	171	NR	NR	8.4	94.6	
		100 mg sitagliptin	170	NR	NR	8.5	93.1	
Brady EM^44^	2014	1.2 mg liraglutide	47	51.5±11.1	NR	7.6±1.1	86.1±16.9	4
		4 mg glimepiride	52	52.2±10.7	NR	7.8±1.0	79±11.2	
Yang W^45^	2011	0.6 mg liraglutide	231	53.5±9.5	7.4±5.4	8.5±1.1	68.6±11.6	3
		1.2 mg liraglutide	233	53.5±9.6	7.5±5.3	8.6±1.1	67.4±11.3	
		1.8 mg liraglutide	234	52.7±9.1	7.2±5.2	8.6±1.1	68.2±11.9	
		4 mg glimepiride	231	53.6±9.7	7.8±6.1	8.5±1.1	68.2±11.9	
Charbonnel B^46^	2013	1.2 mgliraglutide	327	67.6±10.8	8.2±6.2	8.1±0.9	92.1±20.4	3
		100 mg sitagliptin	326	56.9±10	7.6±4.8	8.2±1.1	91±20.5	
Pratley R^40^	2011	1.2 mgliraglutide	135	55.9±9.6	6.0±4.5	8.4±0.8	NR	3
		1.8 mg liraglutide	150	55.0±9.1	6.4±5.4	8.4±0.7	NR	
		100 mg sitagliptin	151	55.0±9.0	6.3±5.4	8.5±0.7	NR	

Abbreviation: NR, not reported; SD, standard deviation.

**Table 2:** Effect comparison between liraglutide added to metformin with other therapies in patients with T2DM.

Pooled changes from baseline between patients treated with combination therapy and other therapies, WMD (95%CI)						
Regimen	Placebo	Sitagliptin	Glimepiride	Dulaglutide	Insulin glargine	NPH
***HbA1c** (**%***)						
1.8 mg liraglutide	−1.09 (−1.11,−1.08)	−0.60 (−0.62, −0.58)	0.00(−0.03, 0.03)	−0.06 (−0.07, −0.05)	−0.24 (−0.26, −0.22)	
1.2 mg liraglutide	−1.10 (−1.13,−1.07)	−0.34 (−0.38, −0.29)	−0.01(−0.11, 0.08)			
0.6 mg liraglutide	−0.80 (−0.83,−0.77)		0.30(0.27, 0.33)			
***Body weight** (**kg***)						
1.8 mg liraglutide	−1.38 (−1.45, −1.31)	−2.52 (−2.71, −2.33)	−2.50 (−2.84, −2.16)	−0.71 (−0.75, -0.67)	−3.40 (−3.47,−3.33)	−1.90 (−2.89,−0.91)
1.2 mg liraglutide		−2.01(−2.78, −1.25)	−2.42 (−2.73, −2.12)			
***FPG** (**mmol*****/*****L***)						
1.8 mg liraglutide	−2.09 (−2.11,−2.07)	−2.43 (−2.49, −2.37)	−0.22 (−0.66, 0.23)	0.03 (0.01, 0.05)	0.24 (0.22, 0.26)	
1.2 mg liraglutide	−2.00 (−2.03,−1.97)	−0.91 (−1.32, −0.05)	−0.10 (−0.52, 0.32)			
0.6 mg liraglutide	−1.50 (−1.53,−1.47)		0.20 (−0.05, 0.45)			
***PPG** (**mmol*****/*****L***)						
1.8 mg liraglutide	−1.91 (−2.07, −1.76)		−0.49 (−1.29, 0.30)	0.13 (0.12, 0.14)	−0.20 (−0.45, 0.05)	−0.70 (−1.57,0.17)
1.2 mg liraglutide	−1.70 (−1.73, −1.67)		−0.11 (−0.73, 0.50)			
0.6 mg liraglutide	−1.10 (−1.13, −1.07)		0.47 (−0.25, 1.18)			
***SBP** (**mm Hg***)						
1.8 mg liraglutide	−2.60 (−2.78, −2.42)	−0.42 (−0.88,0.04)		0.54 (0.43, 0.65)	−4.54 (−4.57, −4.51)	
1.2 mg liraglutide		−1.08 (−4.46, 2.31)	−3.70 (−9.81, 2.41)			
***DBP** (**mm Hg***)						
1.8 mg liraglutide		0.60 (0.52, 0.68)		−0.09 (−0.15, −0.03)		
1.2 mg liraglutide		0.27 (−1.04,1.58)	−5.53 (−9.15, −1.91)			

**Abbreviation**: T2DM, type 2 diabetes mellitus; HbA1c, glycated haemoglobin; FPG, Fasting plasma glucose; PPG, Postprandial plasma glucose; SBP, Systolic blood pressure; DBP, Diastolic blood pressure.

**Table 3:** Summary of the risk ration (RR) of treatment-related adverse events between liraglutide added to metformin and other therapies.

Adverse events	RR	95%CI	*P* value
Nausea	2.26	0.98, 5.25	0.057
Diarrhea	1.98	1.30, 3.00	0.001
Vomiting	1.62	0.94, 2.79	0.080
Dyspepsia	1.31	0.69, 2.52	0.411
Constipation	1.17	0.80, 1.69	0.424
Nasopharyngitis	0.89	0.65, 1.22	0.471
Headache	1.12	0.85, 1.47	0.419
Back pain	1.36	0.64, 2.91	0.430
Deceased appetite	1.25	0.66, 2.36	0.499
Influenza	1.90	0.90, 4.03	0.092
Hypoglycemia	0.33	0.08, 1.44	0.140

Abbreviations: CI, confidence interval.
